# Examining socioeconomic status disparities in facility-based childbirth in Kenya: role of perceived need, accessibility, and quality of care

**DOI:** 10.1186/s12884-022-05111-1

**Published:** 2022-11-02

**Authors:** Ntemena Kapula, Stephen Shiboski, Christine Dehlendorf, Linet Ouma, Patience A. Afulani

**Affiliations:** 1 Department of Epidemiology and Biostatistics, University of California, San Francisco, San Francisco, CA USA; 2 Department of Family and Community Medicine, University of California, San Francisco, San Francisco, CA USA; 3 Department of Obstetrics, Gynecology and Reproductive Sciences, University of California, San Francisco, San Francisco, CA USA; 4 Department of Global Health Sciences, University of California, San Francisco, San Francisco, CA USA; 5 Center for Clinical Research, Kenya Medical Research Institute, Nairobi, Kenya

**Keywords:** Socioeconomic status, Perceived need, Accessibility, Quality of care, Facility delivery, Skilled birth attendance

## Abstract

**Background:**

Giving birth in health facilities with skilled birth attendants (SBAs) is one of the key efforts promoted to reduce preventable maternal deaths in sub-Saharan Africa. However, research has revealed large socioeconomic status (SES) disparities in facility-based childbirth. We seek to extend the literature on the factors underlying these SES disparities. Drawing on the Disparities in Skilled Birth Attendance (DiSBA) framework, we examined the contribution of three proximal factors—perceived need, accessibility, and quality of care—that influence the use of SBAs.

**Methods:**

We used data from a survey conducted in Migori County, Kenya in 2016, among women aged 15–49 years who gave birth nine weeks before the survey (*N* = 1020). The primary outcome is facility-based childbirth. The primary predictors are wealth, measured in quintiles calculated from a wealth index based on principal component analysis of household assets, and highest education level attained. Proposed mediating variables include maternal perceptions of need, accessibility (physical and financial), and quality of care (antenatal services received and experience of care). Logistic regression with mediation analysis was used to investigate the mediating effects.

**Results:**

Overall, 85% of women in the sample gave birth in a health facility. Women in the highest wealth quintile were more likely to give birth in a facility than women in the lowest quintile, controlling for demographic factors (adjusted odds ratio [aOR]: 2.97, 95% CI: 1.69–5.22). College-educated women were five times more likely than women with no formal education or primary education to give birth in a health facility (aOR: 4.96; 95% CI: 1.43–17.3). Women who gave birth in health facilities had higher perceived accessibility and quality of care than those who gave birth at home. The five mediators were estimated to account for between 15% and 48% of the differences in facility births between women in the lowest and higher wealth quintiles.

**Conclusion:**

Our results confirm SES disparities in facility-based childbirth, with the proximal factors accounting for some of these differences. These proximal factors – particularly perceived accessibility and quality of care – warrant attention due to their relationship with facility-birth overall, and their impact on inequities in this care.

**Supplementary Information:**

The online version contains supplementary material available at 10.1186/s12884-022-05111-1.

## Background

High rates of maternal mortality are a critical gender justice issue that has wide-ranging impacts on families, communities, and society. In 2017, about 295,000 women worldwide died due to complications during and after childbirth [1, 2]. Sub-Saharan Africa (SSA) alone accounted for about two-thirds (196,000) of these maternal deaths. Primary contributors to maternal mortality include limited access to both well-equipped maternal health services and skilled birth attendants (SBAs) [3–5]. In SSA, skilled birth attendants are available mainly in health facilities [6], but only 60% of women gave birth in these facilities in 2014–20 [7]. Significant socioeconomic and sociodemographic disparities can be found; in the 2014 Kenya Demographic and Health Survey, 61% of women overall gave birth in health facilities aided by SBAs. However, only 30% of women in the lowest wealth quintile gave birth in health facilities, compared to 93% of women in the highest wealth quintile [7, 8].

Several studies have examined aspects of socioeconomic status (SES) that are associated with deterrence of facility-based childbirth in low-income settings, but few have quantitatively examined contributory factors [9–15]. In Kenya, efforts to encourage more women particularly those from rural and low SES communities to give birth in health facilities – including free maternal health services, health education, and ease of facility access—have been implemented, yet some women still do not give birth in health facilities [16]. Thus, this study aims to extend the literature on factors underlying these SES disparities in facility-based childbirth. The conceptual model informing this research is the Disparities in Skilled Birth Attendance (DiSBA) framework by Afulani and Moyer (2016) [17]. This framework posits that three proximal factors directly affect the use of SBAs: the perceived need for maternal health care, perceived access to maternal health services, and perceived quality of care. Distal factors, such as wealth and education, are hypothesized to indirectly affect the use of SBAs through these proximal factors [17]. The DiSBA framework addresses the effect of SES factors on delays in receiving adequate and appropriate care, which was not explicit in prior models [18–21].

In this study, we seek to examine the role of the proximal factors on facility-based childbirth and to determine whether these factors explain the association between SES predictors—wealth (measured in household wealth quintiles) and education—and facility-based births. We hypothesize that in a population of Kenyan women, those in higher wealth quintiles, with high levels of education, and higher perceptions of quality, need, and access to care are more likely to give birth in health facilities than those with lower wealth, less education, and lower perceptions of the proximal factors. Additionally, we hypothesize that the association between the SES predictors and birth location will be partly explained by these proximal factors.

## Methods

### Data and setting

The data for this analysis are from a 2016 cross-sectional survey conducted in Migori County, Kenya which is described in detail elsewhere [6, 22, 23]. To summarize, data were collected from women aged 15–49 years who delivered in the nine weeks before survey administration. Migori is a predominantly rural county in western Kenya with 8 sub-counties and a population of about one million people [6]. The county has one referral hospital, seven sub-county hospitals, 18 health centers, several dispensaries, and a few faith-based and private health facilities. The estimated total fertility rate for the county is 5.2 children per woman [6].

A multistage sampling approach explained in detail elsewhere [23] was used to select women from each of the 8 sub-counties, with a target of interviewing 200 women from each sub-county. First, Migori County was divided into 8 strata (the 8 sub-counties), within each stratum, 10 community health units were randomly selected. In the Kenyan health service delivery structure, a community health unit is a geographic area set to include approximately 5000 people [23, 24]. From the health unit, women who gave birth within 9 weeks were identified with the help of community health volunteers that were assigned to that community health unit. The study interviews were conducted by trained study field staff in English, KiSwahili, and DhLuo. A total of 1,052 women were interviewed. For this analysis, we used data from 1,020 women with complete information on all the variables of interest. All participants provided informed consent after receiving information about the research from the study team. Participants under the age of 18 years were considered emancipated minors with the ability to give informed consent for themselves because they had recently given birth and were included in the study to represent the population of emancipated minors. Ethical approval for the study was provided by the University of California San Francisco and Kenya Medical Research Institute IRBs.

### Measures

The measures used for the analysis were informed by the DiSBA framework.

### Dependent variable (outcome)

*Health Facility Delivery*. Participants were asked: “Did you deliver in a home or health facility?” From the responses, Home (0) or Health facility (1), we created a binary outcome variable: facility-based delivery.

### Primary predictors

*Household wealth *was measured in quintiles calculated from a wealth index based on the principal component analysis of variables on household assets [25]. The wealth variable was coded as a categorical variable: Poorest (0), Poorer (1), Middle (2), Richer (3), and Richest (4).

*Education. *To determine the highest level of education attained, participants were asked: “What is the highest grade or class that you completed at school?” Response options included the following: No school (0), Attended primary but did not finish (1), Primary (2), Post primary or vocational (3), Secondary (4), College (middle level; 5), and University or above (6). We recoded the education variable by combining smaller categories: No school/primary (0), Post-primary/Vocational/Secondary (1), and College or above (2).

### Potential mediators

We measured the latent variables of perceived need, accessibility, and quality of care using additive indices. Lower scores indicate lower or less positive perceptions, and higher scores denote higher and more positive perceptions.

The *perceived need for maternal health services *variable (Cronbach’s alpha = 0.6) is based on five survey questions, each with a 5-point Likert scale response [17]: Strongly disagree (0), Disagree (1), Neither agree nor disagree (2), Agree (3), and strongly agree (4). The survey statements below attempt to capture the perceived need for maternal health services.

1. “If a woman is healthy, she does not need to deliver in a health facility or with a health provider.”
2. “If a woman has given birth before, she does not need to deliver in a health facility or with a health provider.”
3. “Delivering in a health facility or with a health provider is a sign of weakness.”
4. “Every pregnant woman needs to deliver in a health facility or with a health provider.”
5. “A pregnant woman with no complications can quickly develop complications during labor and delivery.”

The responses to the first three statements were reverse coded, and the responses to all the questions were summed to create a score ranging from 0 to 20.

The *perceived financial access to health services during childbirth *variable (Cronbach’s alpha = 0.7) is based on two survey questions:

1. How easy was it for you to pay for transportation to the health facility?
2. How easy was it for you to get money to buy what you need for your delivery and pay for services at the health facility?

To both questions, women responded: Very easy (0), Easy (1), Difficult (2), and Very difficult (3). We reverse coded both questions and added the responses to create a score ranging from 0 to 6 (2*3).

The *perceived physical access to health services during childbirth *variable (Cronbach’s alpha = 0.7) is based on two survey questions [17]:

1. How easy was it for you to reach this health facility?

Very easy (0), Easy (1), Difficult (2), and Very difficult (3).

2. How do you feel about the amount of time it takes to get to the nearest health facility where deliveries are conducted from your home? Very short (0), Somewhat short (1), Somewhat long (2), and Very long (3).

We reverse coded both questions so that higher perceived physical access produced higher scores. The lowest possible score was 0, and the highest possible score was 6 (2*3).

The *perceived provision of care *variable (Cronbach’s alpha = 0.4) is based on service provision measures during antenatal care, presented as nine survey questions (see Appendix I) asking whether the participants received various services during antenatal care. The responses to the nine questions varied. Five questions were binary: No (0); Yes (1). One question had a three-level response: No (0); Yes, once (1); Yes, more than once (2). Three questions had four-level responses: No (0); Yes, a few times (1); Yes, most times (2); Yes, all of the time (3). The lowest possible score was 0, and the highest possible score was 16 (5*1 + 1*2 + 3*3).

The *perceived experience of care *variable (Cronbach’s alpha = 0.8) is based on 17 experience-of-care survey questions during antenatal visits (see Appendix II in Supplementary file 1). Most women (n = 1019) attended at least one antenatal care appointment during their pregnancy regardless of their childbirth location. The experience-of-care questions capture the provider-patient communication and feeling of dignity and respect. Six questions had binary responses: No (0); Yes (1). Eleven questions had four-level categorical responses: No (0); Yes, a few times (1); Yes, most times (2); Yes, all of the time (3). The lowest possible score was 0, and the highest possible score was 39 (6*1 + 11*3).

### Control variables

Age, marital status, parity (the number of prior births), prior facility-birth, literacy, partner occupation, and paid employment were specified as control variables; they are hypothesized to confound the estimated effect of SES on the delivery outcome.

**Fig. 1:**
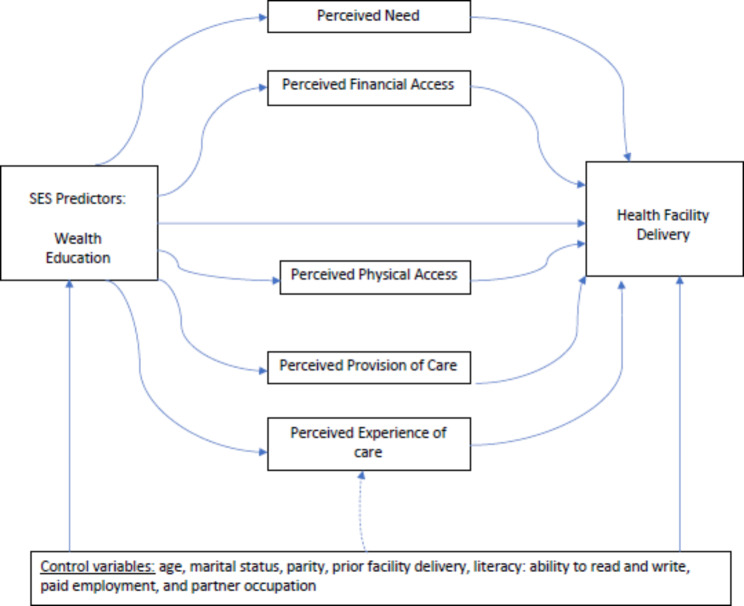
Directed acyclic graph describing the association between SES predictors and delivery facility with control variables to illustrate perception-mediating pathways

### Statistical analysis

Our analysis is informed by the DiSBA conceptual framework and relies on the causal relationships represented in the directed acyclic graph in Fig. 1 [17]. We present descriptive statistics to depict the distributional characteristics of key variables. The characteristics of women who gave birth at a health facility and those who did not were compared using chi-squared tests and t-tests for categorical and continuous variables, respectively. To address possible violations of distributional assumptions we verified results using Fisher’s Exact and Wilcoxon tests.

We first examined the association between wealth (4-level categorical) and facility-based delivery (binary) using logistic regression. The models were constructed by first adding the primary predictor (model 1), followed by the covariates represented as possible confounders in Fig. 1 (model 2), and finally, the mediating predictors (model 3). We followed the same analytic approach in examining mediation of the association between education (3-level categorical) and facility-based delivery.

Standard methods for mediation analysis based on linear models for continuous outcomes correctly estimate the indirect or mediated effect as the difference between the coefficients in models including and excluding the mediating predictor [26]. This approach does not reliably estimate indirect effects for logistic regression related to the non-collapsibility property of odds ratios [27]. Our analysis is based on the Karlson-Holm-Breen (KHB) rescaling method to account for this limitation [27, 28]. The binary logistic model without the mediators (reduced model) was rescaled so that the coefficients of the independent key variables, household wealth and education (c_n_), in the reduced model were comparable to the coefficients of wealth and education in the full model (c_n_’) containing the mediating variables. The indirect effect or mediated effect was calculated as c_n_-c_n_’ for each coefficient, and the total mediated effect percentage was [(c_n_-c_n_’)/c_n_] * 100.

We performed diagnostic tests to ensure the logistic regression models were well-specified (using a goodness-of-fit test) and checked for collinearity between included predictors [29]. We also estimated Bayesian information criterion (BIC) values to compare the overall fit between nested models. A sensitivity analysis was carried out to assess the change in target odds ratio estimates resulting from exclusion of SES covariates. Stata (version 17.0) was used for all analyses [30].

## Results

### Descriptive analyses

Table 1 presents the distribution of the primary predictors, control variables, and mediating variables overall and separately by delivery facility groups. In our sample of 1,020 postpartum women, 85% gave birth in a health facility. Most of the women were between 20 and 29 years old (58%), 79% were married, 69% had more than two prior births, and 62% had previously delivered in a health facility. The analytic sample is almost evenly distributed across wealth quintiles. About 61% of the women had either no education or primary education, 77% were unemployed and 38% had partners involved in agricultural or casual labor. The average perceived need score was 16.7 (SD = 2.7) out of 20; the average perceived financial accessibility score was 2.6 (SD = 1.2) out of 6; the average perceived physical accessibility score was 3.3 (SD = 1.5) out of 6; the average perceived provision of care score was 10.8 (SD = 2.4) out of 16; and the average perceived experience of care score was 25.0 (SD = 7.9) out of 39.

**Table 1:** Univariate and bivariate distributions by delivery facility, N = 1,020

Participant characteristics	Total	Home/Other	Health Facility	
	**N (%)**	**N (%)**	**N (%)**	**P-value**
	1020	148 (15)	872 (85)	
**Age**				0.005
15 to 19 years	174 (17)	16 (9)	158 (91)	
20 to 29 years	592 (58)	81 (14)	511 (86)	
30 to 48 years	254 (25)	51 (20)	203 (80)	
**Marital Status**				0.911
Not Married	217 (21)	32 (15)	185 (85)	
Currently Married	803 (79)	116 (14)	687 (86)	
**Parity (No. of prior births)**				< 0.001
0 to 1 prior births	314 (31)	28 (9)	286 (91)	
2 to 3 prior births	397 (39)	48 (12)	349 (88)	
4 + prior births	309 (30)	72 (23)	237 (77)	
**Prior facility delivery**				0.319
No	389 (38)	51 (13)	338 (87)	
Yes	631 (62)	97 (15)	534 (85)	
**Household Wealth Quintile**				< 0.001
Poorest	247 (24)	58 (24)	189 (76)	
Poorer	231 (23)	43 (19)	188 (81)	
Middle	158 (16)	24 (15)	134 (85)	
Richer	188 (18)	17 (9)	171 (91)	
Richest	196 (19)	6 (3)	190 (97)	
**Highest education**				< 0.001
No school/Primary	616 (61)	124 (20)	492 (80)	
Post-primary/vocational/Secondary	289 (28)	21 (7)	268 (93)	
University/ college or above	115 (11)	3 (3)	112 (97)	
**Literacy: reading and writing very well**				< 0.001
No	241 (24)	59 (25)	182 (75)	
Yes	779 (76)	89 (11)	690 (89)	
**Paid Employment**				0.001
No	782 (77)	129 (17)	653 (83)	
Yes	238 (23)	19 (8)	219 (92)	
**Partner Occupation**				< 0.001
Agricultural labor/Casual labor	393 (38)	70 (18)	323 (82)	
Salaried worker	156 (15)	6 (4)	150 (96)	
Self-employed in petty trade/small scale industry	229 (23)	35 (15)	194 (85)	
Unemployed/homemaker/Other	29 (3)	6 (21)	23 (79)	
No partner	213 (21)	31 (15)	182 (85)	
**Perceived Need Score, mean (SD)** Median (min-max)	16.7 (2.7) 17.0 (4.0–20.0)	16.4 (2.7) 17.0 (7.0–20.0)	16.8 (2.7) 17.0 (4.0–20.0)	0.131
**Perceived Financial Access Score, mean (SD)** median (min-max)	2.6 (1.2) 2.6 (0.0–6.0)	2.3 (1.1) 2.0 (0.0–5.0)	2.7 (1.2) 3.0 (0.0–6.0)	< 0.001
**Perceived Physical Access Score, mean (SD)** median (min-max)	3.3 (1.5) 4.0 (0.0–6.0)	2.6 (1.5) 3.0 (0.0–6.0)	3.5 (1.4) 4.0 (0.0–6.0)	< 0.001
**Perceived Provision of Care Score, mean (SD)** median (min-max)	10.8 (2.4) 11.0 (1.0–16.0)	9.8 (3.0) 10.0 (1.0–16.0)	11.0 (2.3) 11.0 (2.0–16.0)	< 0.001
**Perceived Experience of Care Score, mean (SD)** median (min-max)	25.0 (7.9) 25.0 (0.0–39.0)	23.2 (8.8) 23.5 (5.0–39.0)	25.3 (7.7sta 26.0 (0.0–39.0)	0.004

The percentage of births at health facilities decreases with maternal age, with most facility-based births occurring among women aged 15–19 years (91%). The percentage of facility-based deliveries increases with higher wealth and education levels: 76% of the poorest women delivered at a health facility compared to 97% of the richest and college-educated women. 92% of women with paid employment, and 96% of women whose partners had salaried occupations gave birth in health facilities compared to 83% among those with no paid employment and 79% among those whose husbands were unemployed or homemakers.

The mean scores for perceptions of financial access (2.7; SD = 1.2), provision of care (11.0; SD = 2.3), and experience of care (25.3; SD = 7.7) among women who gave birth in health facilities were higher than among those who gave birth at home [mean scores for perceptions of financial access (2.3; SD = 1.1), provision of care (9.8; SD = 3.0), and experience of care (23.2; SD = 8.8)], and the differences in these perception scores were significant. There was no significant difference between the mean perceived need scores for women who gave birth at a health facility (16.8; SD = 2.7) and those who gave birth at home (16.4; SD = 2.7; p-value = 0.131).

### Regression results

Table 2 details the logistic regression results examining the association between the key predictor wealth and facility-based childbirth. Model 1 is the unadjusted model, model 2 adjusts for confounding covariates, and model 3 further includes mediating variables. The odds of giving birth in a health facility increase with higher household-wealth quintiles in all three models, controlling for other factors, with the highest odds of facility delivery among the richer and richest categories of women (aOR: 2.97, 95% CI: 1.69–5.22).

**Table 2:** Logistic regression models for dependence of facility delivery on wealth, N = 1,020

	Odds of delivering in a Health facility: OR [95% CI]		
	**Model 1** Unadjusted wealth model	**Model 2** Wealth + covariates	**Model 3** Wealth + covariates + mediators
**Household Wealth**			
Poorest	**Ref.**		
Poorer	1.34 [0.86, 2.09]	1.30 [0.82, 2.06]	1.19 [0.73, 1.93]
Middle	1.71* [1.01, 2.90]	1.42 [0.82, 2.45]	1.18 [0.67, 2.08]
Richer/ Richest	4.82*** [2.88, 8.05]	2.97*** [1.69, 5.22]	2.60** [1.44, 4.69]
**Age**			
15 to 19 years		1.63 [0.82, 3.23]	1.78 [0.88, 3.59]
20 to 29 years		**Ref.**	
30 to 48 years		0.78 [0.49, 1.24]	0.85 [0.52, 1.38]
**Parity (no. of prior births)**			
0–1 prior births		1.11 [0.60, 2.04]	1.05 [0.56, 1.99]
2 to 3 prior births		**Ref.**	
4 + prior births		0.66 [0.42, 1.05]	0.73 [0.45, 1.19]
**Paid employment**			
No		**Ref.**	
Yes		1.93* [1.12, 3.31]	1.64 [0.93, 2.87]
**Literacy: ability to read and write**			
No		**Ref.**	
Yes		1.56* [1.04, 2.34]	1.48 [0.96, 2.27]
**Partner occupation**			
Agricultural labor/Casual labor		**Ref.**	
Salaried worker		2.69* [1.10, 6.53]	2.55* [1.03, 6.34]
Self-employed in petty trade/small scale industry		0.99 [0.62, 1.57]	0.87 [0.54, 1.40]
Unemployed/homemaker/Other		0.55 [0.21, 1.48]	0.65 [0.23, 1.82]
No partner		0.80 [0.47, 1.35]	0.78 [0.45, 1.36]
**Perceived need**			0.99 [0.92, 1.06]
**Perceived financial access**			0.95 [0.78, 1.16]
**Perceived physical access**			1.44*** [1.23, 1.69]
**Perceived provision of care**			1.19*** [1.09, 1.30]
**Perceived experience of care**			0.98 [0.95, 1.01]
Constant	3.26 [2.43, 4.37]	2.81 [1.70, 4.64]	0.37 [0.09, 1.57]
Observations	1020	1020	1020
Pseudo-R-squared	0.05	0.10	0.15
BIC	827.7	857.6	853.5

*Model 1 is unadjusted wealth model, Model 2 is the adjusted model of wealth and covariates, Model 3 is the adjusted model of wealth + covariates and mediators*

* p < 0.05, ** p < 0.01 and ***p < 0.001

Table 3 presents logistic regression results on the effect of education on facility delivery. College-educated women have higher odds of giving birth in a health facility than their peers with no school or primary education (aOR: 4.96, 95% CI: 1.43–17.3).

**Table 3:** Logistic regression models for dependence of facility delivery on education, N = 1,020

	Odds of delivering in a Health facility: OR [95% CI]		
	**Model 1** Unadjusted Education model	**Model 2** Education + covariates	**Model 3** Education + covariates + mediators
**Education**			
No school/Primary	**Ref.**	**Ref.**	
Post-primary/vocational/Secondary	3.22*** [1.98, 5.23]	2.21** [1.28, 3.82]	2.24** [1.29, 3.91]
University/ college or above	9.41*** [2.94, 30.1]	4.96* [1.43, 17.3]	4.77* [1.34, 16.89]
**Age**			
15 to 19 years		1.99 [0.98, 4.05]	2.25* [1.07, 4.72]
20 to 29 years		**Ref.**	**Ref.**
30 to 48 years		0.76 [0.47. 1.21]	0.82 [0.50, 1.33]
**Parity (no. of prior births)**			
0–1 prior births		0.95 [0.50, 1.79]	0.88 [0.45, 1.70]
2 to 3 prior births		**Ref.**	
4 + prior births		0.72 [0.45, 1.14]	0.80 [0.49, 1.30]
**Paid employment**			
No		**Ref.**	
Yes		1.88* [1.09, 3.24]	1.63 [0.92, 2.87]
**Literacy: ability to read and write**			
No		**Ref.**	
Yes		1.62* [1.08, 2.41]	1.50 [0.99, 2.29]
**Partner occupation**			
Agricultural labor/Casual labor		**Ref.**	
Salaried worker		2.36 [0.96, 5.81]	2.20 [0.87, 5.53]
Self-employed in petty trade/small scale industry		1.04 [0.65, 1.64]	0.89 [0.55, 1.43]
Unemployed/homemaker/Other		0.55 [0.20, 1.47]	0.67 [0.24, 1.86]
No partner		0.78 [0.46, 1.31]	0.76 [0.44, 1.32]
**Perceived need**			0.98 [0.92, 1.06]
**Perceived financial access**			0.97 [0.80, 1.18]
**Perceived physical access**			1.45*** [1.24, 1.69]
**Perceived provision of care**			1.18*** [1.09, 1.29]
**Perceived experience of Care**			0.98 [0.95, 1.01]
Constant	3.97 [3.26, 4.83]	3.18 [2.00, 5.06]	0.41 [0.09, 1.73]
Observations	1020	1020	1020
Pseudo-R-squared”	0.06	0.1	0.15
BIC	817.8	852.2	844.9

*Model 1 is unadjusted education model, Model 2 is the adjusted model of education and covariates, Model 3 is the adjusted model of education + covariates and mediators*

* p < 0.05, ** p < 0.01 and ***p < 0.001

In Tables 2 and 3, we find that the odds of a facility delivery decrease with increasing age and parity. Women who can read and write well and those with paid employment are more likely to give birth in a health facility.

Among the five mediators assessed, perceived physical accessibility, and perceived provision of care were significantly associated with higher odds of giving birth in a health facility in the multivariable model. The other perception variables (perceived experience of care, perceived need, and perceived financial accessibility) were not significant in the multivariate model.

### Mediation assessment

Table 4 presents the mediation results. Collectively, our five mediators (perceived need, financial accessibility, physical accessibility, provision of care, and experience of care) account for 28% (standard error [SE]: 43.3%, p-value = 0.52) of the difference between women in the poorer and poorest quintiles, 48% (SE: 32%, p-value = 0.13) of the difference between women in the middle and poorest quintiles, and 15% (SE: 10%, p-value = 0.12) of the difference between the richer or richest women and the poorest. All five predictors demonstrate a mediated effect of less than 10% on the association between education and facility delivery across all levels of education.

**Table 4:** Estimated mediation of the effect on facility delivery by perception variables

	Mediated Effect Coef. [Std. Err]	P-Value	% Of total effect mediated					
			**Perceived Need**	**Perceived Financial Accessibility**	**Perceived Physical Accessibility**	**Perceived Provision of Care**	**Perceived Experience of Care**	**All 5 mediators**
**Household Wealth**								
Poorest	**Ref.**							
Poorer	0.07 [0.10]	0.52	0.12 [1.05]	10.3 [19.4]	55.2 [23.0]	19.0 [15.8]	1.81 [5.48]	27.9 [43.3]
Middle	0.15 [0.10]	0.13	0.35 [1.55]	5.80 [11.0]	48.8 [19.9]	9.03 [13.1]	3.28 [5.25]	48.4 [32.1]
Richer/ Richest	0.17 [0.11]	0.12	0.04 [0.24]	2.59 [4.88]	15.0 [5.31]	3.17 [3.30]	0.71[1.26]	14.9 [9.60]
**Education**								
No school/Primary	**Ref.**							
Post-primary/ vocational/Secondary	0.05 [0.09]	0.62	0.24 [0.69]	0.55 [1.82]	2.68 [5.03]	5.36 [3.98]	1.95 [2.05]	5.30 [10.7]
College or above	0.10 [0.10]	0.33	0.45 [1.12]	0.24 [0.84]	0.84 [4.01]	4.52 [5.32]	1.11 [1.39]	5.78 [5.9]
*Standard Error in square brackets* *‘khb’ rescaling method used to determine mediation*								

The perceived physical accessibility is the largest contributor to the mediated effect, followed by the perceived provision of care. For example, 55% of the total mediated effect is due to the perceived physical accessibility in the association between wealth and facility delivery between women in the poorer and poorest quintiles, and 19% of the total mediated effect is due to the perceived provision of care.

### Sensitivity analysis

We conducted a sensitivity analysis to assess changes in estimated odds ratios of primary predictors of wealth and education resulting from excluding SES variables (literacy, paid employment, and partner occupation) from the regression model and mediation analysis, leaving the maternal factors of age and parity. We found that the estimated odds ratios of facility-based delivery at all levels of wealth and education were elevated when other SES variables were not included. These changes in estimates could reflect the confounding effects of the excluded variables and/or the non-collapsibility property of odds ratios [31].

In the mediation analysis, the total mediated effect varied slightly across all wealth quintiles when the other SES control variables (literacy, paid employment, and partner occupation) were excluded from the mediation analysis. The total mediated effect of all five perception variables among the poorer compared to the poorest women increased by 4%, while it decreased by 0.22% among women in the middle-wealth quintile compared to the poorest women. The total mediated effect increased by 5% among women with a post-primary/vocational or secondary education and by 6% among college-educated women compared to women with no schooling or primary education when the other SES factors were excluded from the mediation analysis (results not shown).

## Discussion

In this paper, we tested the DiSBA conceptual framework for the underlying causes of socioeconomic disparities in skilled birth attendance using a sample from rural Kenya. As expected, we found that higher wealth and education were associated with facility births, and that the mediators of the perceived access and quality of care variables, perceived physical access to the nearest health facility, and the perceived provision of care variables had accounted for between 15% and 48% of the wealth differences and about 10% of the education difference in facility births.

Our findings on the associations between women’s education, wealth, age, parity, literacy, and paid employment with skilled birth attendance are consistent with studies that examined the determinants of facility-based childbirth aided by skilled birth attendants [16, 18, 32, 33]. Consistent with our findings, most studies find that with increasing level of education and wealth as well as having paid employment, women were more likely to give birth in a health facility with skilled birth attendants and with increasing age and parity, women were less likely to give birth in health facilities. We also found that the odds of delivering in a health facility were higher with increasing perceived physical access. This is consistent with the premise of DiSBA framework. The findings align with several studies indicating that the ability to physically reach the nearest health facility is a key predictor of a health facility delivery in Kenya [9, 13, 34, 35]. In a study conducted by Moindi et al. [35] in Kilifi County, Kenya, only a long distance (≥ 10Kms) to the nearest delivery facility was associated with a higher risk of delivery at home in the multivariate analysis [35]. The lack of association with financial accessibility is likely due to the free maternal health care program in Kenya [36].

Our findings on perceived quality of and receipt of care are partially consistent with prior literature. For example, a study by Kifle et al. that examined the factors influencing the choice of delivery place in Eritrea found that women with good perceptions of the quality of care they received (measured as satisfied with service provision of antenatal and delivery care) were ten times more likely to deliver in a health facility [37]. In the multivariate analysis, we find this association only with the service provision variable. We created the perceived provision of care and experience of care variables based on services received and experiences during antenatal care based on the assumption that a woman’s perception of maternal health services prior to her encounter with the health facility childbirth is partially influenced by her antenatal care experience. Several studies in Kenya have found that women who attend the recommended four or more antenatal visits are more likely to give birth in a health facility; this may be because pregnant women become familiar with maternal health care systems and receive education on the importance of a facility delivery [9, 10, 38, 39]. If women believe they will receive adequate care during childbirth in a health facility, based on their antenatal care experience and birth preparation education, women may regard a health facility delivery with an SBA more highly. A potential reason why the association between facility-based childbirth and perceived antenatal experience of care was not significant in the multivariate analysis is the widespread perceptions of disrespect and abuse during facility-based childbirth [40]. These widespread negative community perceptions of the facility-based birth experience might outweigh women’s perceptions of how their individual interactions with providers during antenatal care may influence their childbirth experience.

The lack of significant association with perceived need in both bivariate and multivariate analyses may be due to the generally high perceived need in the sample, which is likely due to prevalent education on the benefits of facility birth [41–43]. Additionally, even women with high perceived need may not use a service if it is not accessible to them. Further, intention does not always translate to actions. In an innovative prospective study, Creanga et al [44]. examined pregnant women’s intentions and subsequent behaviors regarding maternal health utilization during antenatal, childbirth, and postnatal phases. They found that about 98% of pregnant women intended to deliver in a health facility, but only about 77% delivered in a health facility [44]. Factors cited for this difference include the distance to the health facility; some women give birth before reaching the delivery facility, either en route or at home. Additionally, women may have indicated the intention to deliver in health facilities during the study due to social desirability bias.

To our knowledge, this is one of the first studies to test the assumptions of the DiSBA model. The prior analysis using this model, which was based on data from Ghana, was limited by the absence of data on most of the proximal factors. Perceived accessibility and quality of care appear to be the most important proximal factors in Migori county, and therefore should be prioritized in efforts to improve facility births in the county. The most important proximal factors may however differ in other settings, warranting analysis to better understand the factors contributing to disparities in different settings to inform context specific interventions. More studies are also needed to test the assumptions of the DiSBA model in various low-and middle-income countries using more standardized measurement of the proximal factors.

## Strengths and limitations

This study has its limitations; the main one is that the perception measures used are not based on validated tools and so may not adequately capture all the dimensions of the constructs being assessed. There is a need for a more systematic process for developing validated scales to measure these proximal factors. Additionally, all measures are based on self-report, with recall and social desirability bias as potential limitations [45]. Furthermore, the cross-sectional study design limits causal inference, and we can only depict associations. Finally, this was a secondary analysis based on a larger study on perceived quality of maternity care in western Kenya where a large proportion of women enrolled in the study gave birth in health facilities and this sample may not be representative. The findings may not also be generalizable to other settings given that the data are from one rural county in Kenya. Nonetheless, this study makes an important contribution to the literature. A major strength of this paper is the use of a conceptual framework to inform the analysis. It is also one of the first studies to test the DiSBA framework in a different context and adds to the body of literature quantitatively examining the factors underlying the SES disparities in facility-based birth.

## Conclusion

This study applied the DiSBA framework to examine the factors contributing to SES disparities in facility-based births in a rural county Kenya. Our results suggest that proximal factors—particularly perceived accessibility and quality of maternal healthcare —directly affect where a woman gives birth, thus warranting our attention. Potential ways to reduce SES disparities in skilled birth attendance include enhancing perceptions of maternal health services through improved physical and financial access and the provision of high-quality respectful maternity care. The proximal factors may differ in other settings; hence it would be beneficial to apply the DiSBA framework in other settings to test its assumptions and inform context specific interventions.

## Electronic supplementary material

Below is the link to the electronic supplementary material.

**Supplementary Material 1**

**Supplementary Material 2**

## Data Availability

The data analyzed during this study are included in this research manuscript as a supplementary document (Supplementary file 2).

## Abbreviations

DiSBA: Disparities in Skilled Birth Attendance.
SBA: Skilled Birth Attendant.
SES: Socio-Economic Status.
aOR: adjusted Odds Ratio.
SE: Standard Error.
