# Supplementary material for: Examining socioeconomic status disparities in facility-based childbirth in Kenya: role of perceived need, accessibility, and quality of care
Source: BMC Pregnancy Childbirth. 2022 Nov 2;22:804. doi: 10.1186/s12884-022-05111-1 (PMC9628025; doi:10.1186/s12884-022-05111-1)
Supplement: Supplementary file 1 — Supplementary Material 1 [file 12884_2022_5111_MOESM1_ESM.docx]

**APPENDIX I: Survey Questions** included to form the **Perceived Provision of Care** construct

As part of your antenatal care during this pregnancy, were any of the following done:

85 Was your height measured?

0 No

1 Yes

8 Don't know or can't remember

86 Were you weighed? (If yes) Were you weighed all the time, most of the time, or a few times?

0 No, Never

1 Yes, A Few Times

2 Yes, Most Of The Time

3 Yes, All The Time

8 Don't Know Or Can't Remember

89 Was your blood pressure taken? (If yes) Was your blood pressure taken all the time, most of the time, or a few times?

0 No, Never

1 Yes, A Few Times

2 Yes, Most Of The Time

3 Yes, All The Time

8 Don't Know Or Can't Remember

91 Did you give a urine sample? (If yes) Did you give a urine sample all the time, most of the time, or a few times?

0 No, Never

1 Yes, A Few Times

2 Yes, Most Of The Time

3 Yes, All The Time

8 Don't Know Or Can't Remember

93 Did you give a blood sample? (If yes) Did you do a blood test once or more than once?

0 No

1 Yes, Once

2 Yes, More than once

8 Don't know or can't remember

95 During this pregnancy, were you given an injection in the arm to prevent the baby from getting tetanus, that is, convulsions after birth?

0 No

1 Yes

8 Don't know or can't remember

96 During this pregnancy, were you given, or did you buy any iron tablets or iron syrup?

0 No

1 Yes

8 Don't know or can't remember

97 During this pregnancy, were you given, or did you buy any drug for intestinal worms?

0 No

1 Yes

8 Don't know or can't remember

98 During this pregnancy, were you given any drugs to keep you from getting malaria?

0 No

1 Yes

8 Don't know or can't remember

**APPENDIX II: Survey Questions** included to form the **Perceived experience of care** construct

87 Were you told the results after you were weighed? (If yes) Were you told the results all the time, most of the time, or a few times?

0 No, Never

1 Yes, A Few Times

2 Yes, Most Of The Time

3 Yes, All The Time

8 Don't Know Or Can't Remember

90 Were you told the results after your blood pressure was taken? (If yes) Were you told the results all the time, most of the time, or a few times?

0 No, Never

1 Yes, A Few Times

2 Yes, Most Of The Time

3 Yes, All The Time

8 Don't Know Or Can't Remember

92 Were you told the results of the urine test? (If yes) Were you told the results all the time, most of the time, or a few times?

0 No, Never

1 Yes, A Few Times

2 Yes, Most Of The Time

3 Yes, All The Time

8 Don't Know Or Can't Remember

94 Were you told the results of the blood test? (If yes) Were you told the results all the time, most of the time, or a few times?

0 No, Never

1 Yes, A Few Times

2 Yes, Most Of The Time

3 Yes, All The Time

8 Don't Know Or Can't Remember

100 During any of your antenatal care visit(s):

Were you told about the signs of pregnancy complications?

0 No

1 Yes

8 Don't know or can't remember

101 Were you told where to go if you had any complications?

0 No

1 Yes

8 Don't know or can't remember

104 Were you ever told what to expect in the course of your pregnancy and delivery?

0 No

1 Yes

8 Don't know or can't remember

105 During any of your antenatal care visit(s):

Did health provider ever talk to you about making prior arrangements for how to get to the health facility when you go into labor?

0 No

1 Yes

8 Don't know or can't remember

106 Did a health provider ever talk to you about what to eat or how to eat well?

0 No

1 Yes

8 Don't know or can't remember

107 Were you given any information or counseled about breast feeding?

0 No

1 Yes

8 Don't know or can't remember

108 Thinking about all your antenatal care visits, Did you feel the doctors, nurses or other staff treated you with respect? (If yes) Will you say you were treated with respect all the time, most of the time, or only a few times?

0 No, never

1 Yes, a few times

2 Yes, most of the time

3 Yes, all the time

8 Don't know or can't remember

109 Thinking about all your antenatal care visits, Did you feel the doctors, nurses or other staff treated you in a friendly manner? (If yes) Will you say you were treated in a friendly manner all the time, most of the time, or only a

few times?

0 No, never

1 Yes, a few times

2 Yes, most of the time

3 Yes, all the time

8 Don't know or can't remember

110 Thinking about all your antenatal care visits, Do you feel you could discuss your problems with the doctors, nurses or other providers, without others not involved in your care overhearing your conversations? (If yes) Will you say you had privacy all the time, most of the time, or only a few times?

0 No, never

1 Yes, a few times

2 Yes, most of the time

3 Yes, all the time

8 Don't know or can't remember

111 Thinking about all your antenatal care visits, Did you feel you understood the purpose of any tests you were asked to do? (If yes) Will you say this was all the time, most of the time, or only a few times?

0 No, never

1 Yes, a few times

2 Yes, most of the time

3 Yes, all the time

8 Don't know or can't remember

112 Thinking about all your antenatal care visits, Did you feel you understood the purpose of any medicines you were given? (If yes) Will you say this was all the time, most of 8 Don't know or can't remember the time, or only a few times?

0 No, never

1 Yes, a few times

2 Yes, most of the time

3 Yes, all the time

113 Thinking about all your antenatal care visits, Did you feel you could ask the doctors, nurses or other 1 Yes, a few times staff at the facility any questions you had? (If yes) Will you say this was all the time, most of the time, or only a few times?

0 No, never

2 Yes, most of the time

3 Yes, all the time

8 Don't know or can't remember

114 Thinking about all your antenatal care visits, Did the doctors, nurses or other staff at the facility ask you if you had any questions? (If yes) Will you say this was all the time, most of the time, or only a few times?

0 No, never

1 Yes, a few times

2 Yes, most of the time

3 Yes, all the time

8 Don't know or can't remember
